# Differentiating head and neck carcinoma from lung carcinoma with an electronic nose: a proof of concept study

**DOI:** 10.1007/s00405-016-4038-x

**Published:** 2016-04-16

**Authors:** Michel R. A. van Hooren, Nicoline Leunis, Dirk S. Brandsma, Anne-Marie C. Dingemans, Bernd Kremer, Kenneth W. Kross

**Affiliations:** 1 Department of Otorhinolaryngology, Head and Neck Surgery, Maastricht University Medical Center, PO Box 5800, 6202 AZ Maastricht, The Netherlands; 2 Department of Pulmonology, Maastricht University Medical Center, Maastricht, The Netherlands

**Keywords:** Volatile organic compounds, Electronic nose, Head and neck carcinoma, Lung carcinoma, Diagnosis

## Abstract

Disease specific patterns of volatile organic compounds can be detected in exhaled breath using an electronic nose (e-nose). The aim of this study is to explore whether an e-nose can differentiate between head and neck, and lung carcinoma. Eighty-seven patients received an e-nose measurement before any oncologic treatment. We used PARAFAC/TUCKER3 tensor decomposition for data reduction and an artificial neural network for analysis to obtain binary results; either diagnosed as head and neck or lung carcinoma. Via a leave-one-out method, cross-validation of the data was performed. In differentiating head and neck from lung carcinoma patients, a diagnostic accuracy of 93 % was found. After cross-validation of the data, this resulted in a diagnostic accuracy of 85 %. There seems to be a potential for e-nose as a diagnostic tool in HNC and lung carcinoma. With a fair diagnostic accuracy, an e-nose can differentiate between the two tumor entities.

**Electronic supplementary material:**

The online version of this article (doi:10.1007/s00405-016-4038-x) contains supplementary material, which is available to authorized users.

## Introduction

Head and neck squamous cell carcinoma (HNSCC) and lung cancer have a major impact on global health. In 2012, a worldwide estimate of 686,000 and 1,825,000 new cases of head and neck and lung cancer were diagnosed each year, respectively, with an estimated death rate of 5 and 19 %, respectively [1]. Early diagnosis improves prognosis considerably [2–4], however, diagnosis of HNSCC and lung cancer is rather invasive, since the gold standard is histopathology with biopsies which have to be obtained through bronchoscopy, or endoscopy of the head and neck area. Therefore, a non-invasive diagnostic tool might be useful in this population. Moreover, differentiating between primary lung malignancies and metastases to the lung of head and neck origin could aid in therapy decision making.

In the past decades, Volatile Organic Compounds (VOC’s) were broadly investigated as diagnostic biomarkers in medicine. Using sniffer dogs, gas chromatography, mass spectroscopy or pattern recognition, VOC’s can be detected in exhaled breath, feces or urine to diagnose various diseases [5–7]. One device to investigate VOC patterns is an electronic nose (e-nose). In exhaled breath, Dragonieri et al. [8] compared VOC patterns of patients with lung cancer, COPD, and healthy controls using an e-nose and concluded that, with an accuracy of 85–90 %, VOC patterns of these groups differ significantly. For HNSCC, our group [7] evaluated VOC patterns in exhaled breath of 36 HNSCC patients and 23 patients without malignant disease with an e-nose and revealed a 90 % sensitivity and 80 % specificity in diagnosing HNSCC.

Peng et al. [9] used an e-nose to differentiate between lung, breast, prostate and colon carcinoma in a proof of concept study with VOC pattern recognition. They concluded that different cancer types have different VOC patterns. To our knowledge, no studies have been published regarding the possibility of VOC pattern recognition to differentiate HNSCC and lung carcinoma. As HNSCC and lung carcinoma are both part of the respiratory tract and share some risk factors like smoking, radiation exposure and exposure to certain carcinogens like asbestos, it would be interesting to know whether an e-nose can discriminate between both cancer types [10, 11]. Moreover, as a synchronous second primary lung tumor occurs in 0.8 % of cases in HNSCC [12], an e-nose could possibly help detecting second primary tumors or differentiate between metastases or primary malignancies. Therefore, the purpose of this study is to determine whether VOC pattern recognition can discriminate between breath of primary HNSCC patients and primary lung carcinoma patients using a non-invasive e-nose.

## Materials and methods

### Participants

For this study, patients with suspect primary HNSCC or lung carcinoma were recruited in a tertiary care referral hospital; the Maastricht University Medical Centre (MUMC). Exclusion criteria were: age under 18 years, current tracheostomy, having had any treatment for current tumor, and a history of any other form of cancer. Patients were also excluded if they did or could not complete the full 5 min of measurement or were unable to endure a nose clip during measurement to promote oral breathing through the e-nose. Cutaneous tumors or malignancies of the salivary glands were excluded in this study. Tumor characteristics and medical history were collected from the clinical records.

Breath analysis can be influenced by internal and external pollution of the exhaled breath [13]. To minimize external pollution of ambient air in the room in which the measurement is performed, the lungs are rinsed with clean filtered air during measurement. Minimizing internal factors is more difficult since local factors in the gastro-intestinal and upper and lower respiratory tract can contribute to the VOC’s in the exhaled air [13]. Moreover, metabolites in the blood due to, e.g., starvation or oxidative stress due to smoking can be excreted in urine or exhaled breath [13–15]. Therefore, we documented metabolic fasting state and smoking habits in this study to take into consideration in this study. Metabolic fasting state was defined as no food or drinks 4 h before the measurement with the exception of two units of clear liquids 2 h before the measurement. Non-smoking was defined as no smoking in the previous month.

Side- or adverse-effects during or shortly after measurement were documented. Oral informed consent was obtained from all patients. The study protocol was approved by the medical ethics committee in accordance of the declaration of Helsinki.

### Study design

All patients were asked to in- and exhale through the e-nose for 5 min. Before the measurement, patients were instructed to get acquainted with the e-nose by performing some test in- and exhalations. A nose clip was placed on the nose to avoid entry of non-filtered air. Patients were instructed to enclose the lips over the mouthpiece at all times.

E-nose measurements were performed in parallel with the regular diagnostic work-up. However, participants did not receive any diagnostic results from the e-nose measurement. The routine diagnostic work-up was based on national cancer guidelines from the Dutch Head and Neck Society and independent of e-nose measurements. E-nose outcomes were compared to histopathology of biopsies.

## Materials

The e-nose used in this study, (Aeonose, the eNose Company, Zutphen, the Netherlands), consists of three micro hotplate metal oxide sensors (AS-MLV sensors, Applied Sensors GmbH). During the measurement the hotplate will be periodically heated and cooled between 260 and 340 °C in 32 steps, during which they are exposed to the exhaled breath. The reduction and oxidation (redox) reactions of the VOC’s at the surface of the metal oxide sensors result in a change in conductivity of the sensors. These changes in conductivity over time, with altering temperature create a unique pattern of redox-reactions of the VOC’s.

A measurement cycle lasts for about 15 min, of which 5 min of in- and exhalation by the patient takes place. Patients breath through the Aeonose via a disposable mouthpiece with a high efficiency particulate arrestance (HEPA) filter to protect the Aeonose against contamination by, e.g., bacteria and viruses. After this, patients inhale through a carbon filter and a valve to filter the environmental air of disturbing VOC’s, which may tamper with the measurement.

The first 2 min of the measurement cycle is used to rinse patient's lungs with clean filtered air and remove dead air space. Rinsing the lungs minimizes the possible external confounding factors of the air in the room where the measurement is being performed. During the next 3 min, patient's exhaled breath is analyzed by the sensors. A small pump ensures a constant flow of exhaled air passing the sensor surface and a Tenax tube. The combination of sensors and the Tenax tube ensures an optimal detection of the VOC’s present, even at a low VOC concentration. After the patient has put the Aeonose down, regeneration of the sensors takes place using filtered environmental air passage through the carbon filter and subsequently the Tenax tube is heated to detect possible low concentrated VOC’s in the exhaled breath. Finally, the sensors are regenerated again using filtered air.

### Statistical analysis

Differences in baseline characteristics were determined using independent sample t test, Fisher’s exact test, or Pearson’s Chi-square test. All statistical analyses were performed using IBM SPSS Statistics for Windows, Version 23.0 (Armonk, NY: IBM Corp.).

Each e-nose measurement results in 32 (temperature) times 36 (measurement cycles) times 3 (sensor) data points, which can be regarded as a 3-dimensional multi-way dataset of temperature versus time versus sensor type, respectively. First, the data are being compressed through PARAFAC/TUCKER3 tensor decomposition. Secondly, the remaining vectors representing the coded patient information are analyzed by an artificial neural network (ANN). This is being executed for a number of data scaling options resulting in 21 different designs for separating ‘sick’ and ‘healthy’ individuals. The ANN is per protocol based in this proof of concept study to exclude possible imperfections of the data. Patients were excluded from the analysis when being falsely diagnosed in 85 % of used designs. Data compression and ANN have been integrated in a proprietary software package (Aethena, the eNose Company, Zutphen, the Netherlands). The binary results are presented in a scatterplot and a receiver operating characteristic curve (ROC-curve). Matthews Correlation Coefficients (MCC) were calculated to measure the quality of binary classifications and 95 % confidence intervals (CI) were given.

All data were premarked with the diagnosis of either HNSCC or lung carcinoma when processed in Aethena. A best fit model of the data was calculated. To predict the fit of a model for future undefined breath samples, cross-validation of the data was performed using a leave-one-out method. This internal validation prevents to a high extent fitting of data on artifacts instead of breath profile classifiers.

## Results

### Patient characteristics

**Fig. 1:**
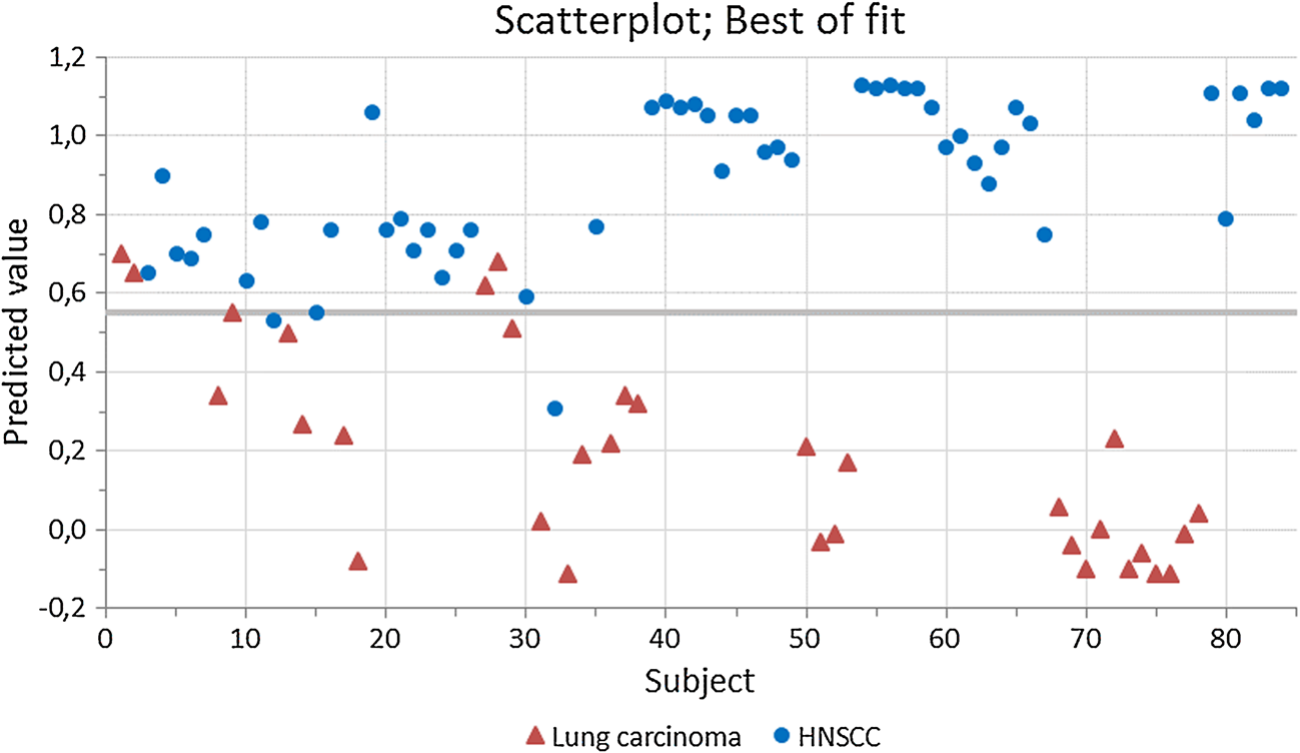
Scatterplot of best of fit of data

**Fig. 2:**
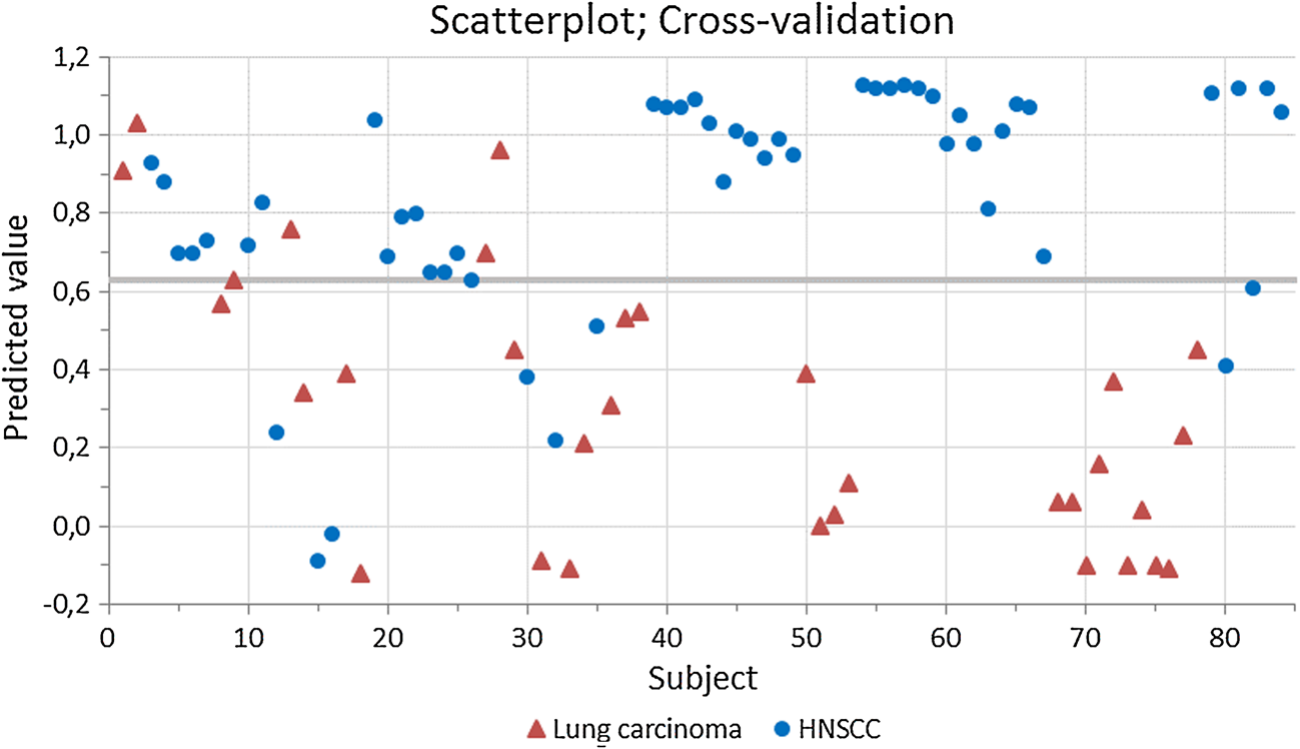
Scatterplot of cross-validation of the data

This study included eighty-seven patients with histopathological proven HNSCC (*N* = 53) or lung carcinoma (*N* = 34). Three patients were excluded from analysis, since they were assigned to the wrong group in over 85 % of the tested designs by the per-protocol-based ANN. These three patients included a T2N0M0 squamous cell carcinoma of the oral cavity, and two stage IV lung carcinoma patients (adenocarcinoma and neuroendocrine tumor). Baseline characteristics of the included patients are listed in Table 1. Only food intake in the past 4 h revealed significant baseline group differences. Five Aeonoses were used to perform the measurements to exclude possible machine-bound confounding factors. Tumor sites of included HNSCC patients were oral cavity (*N* = 15), oropharynx (*N* = 13), nasopharynx/nasal cavity (*N* = 2), hypopharynx (*N* = 3), and larynx (*N* = 19) and all patients were diagnosed with squamous cell carcinoma. Of the patients with lung carcinoma, five patients were diagnosed with squamous cell carcinoma, eighteen with adenocarcinoma, three with small cell carcinoma, and six with other malignancies (malignant mesothelioma, neuroendocrine tumor). Using Pearson’s Chi-square we found significant (0.000) baseline differences between histopathology of included head and neck to lung carcinoma patients. The distribution of tumor-stage amongst both groups is displayed in Table 1. Significant baseline differences between both groups were found, where lung carcinoma patients usually have more advanced disease compared to HNSCC patients. Two patients with lymph node metastasis of initially unknown primary tumors were found. Most individual patient characteristics including TNM-stage are listed in the online resources, where patient numbers correspond with the numbers in Figs. 1 and 2 (Online Resource 1).

**Table 1:** Baseline characteristics of included patients

	Lung	Head and Neck	*p* value
No. of patients	32	52	
Age (years)	65	63	0.362#
Sex (male)	20	43	0.068^
Food intake <4 h (Yes)	30	28 (12 unknown)	0.001*
Currently smoking	13	32	0.074^
Aeonose serial number			0.060*
259	9	6	
309	2	14	
315	4	11	
362	9	11	
379	8	10	
Tumor stage			0.005*
I	4 (13 %)	16 (32 %)	
II	1 (3 %)	10 (20 %)	
III	10 (32 %)	5 (10 %)	
IV	16 (52 %)	19 (38 %)	
Missing data	1	2	

*No* number

* Pearson Chi-square

^ Fisher’s exact test

# Independent *t* test

Three patients reported a feeling of dyspnea at the end of the measurement and shortly after. No additional follow-up or intervention was needed for all three patients. No other side- or adverse-effects were reported.

### Data analysis

**Fig. 3:**
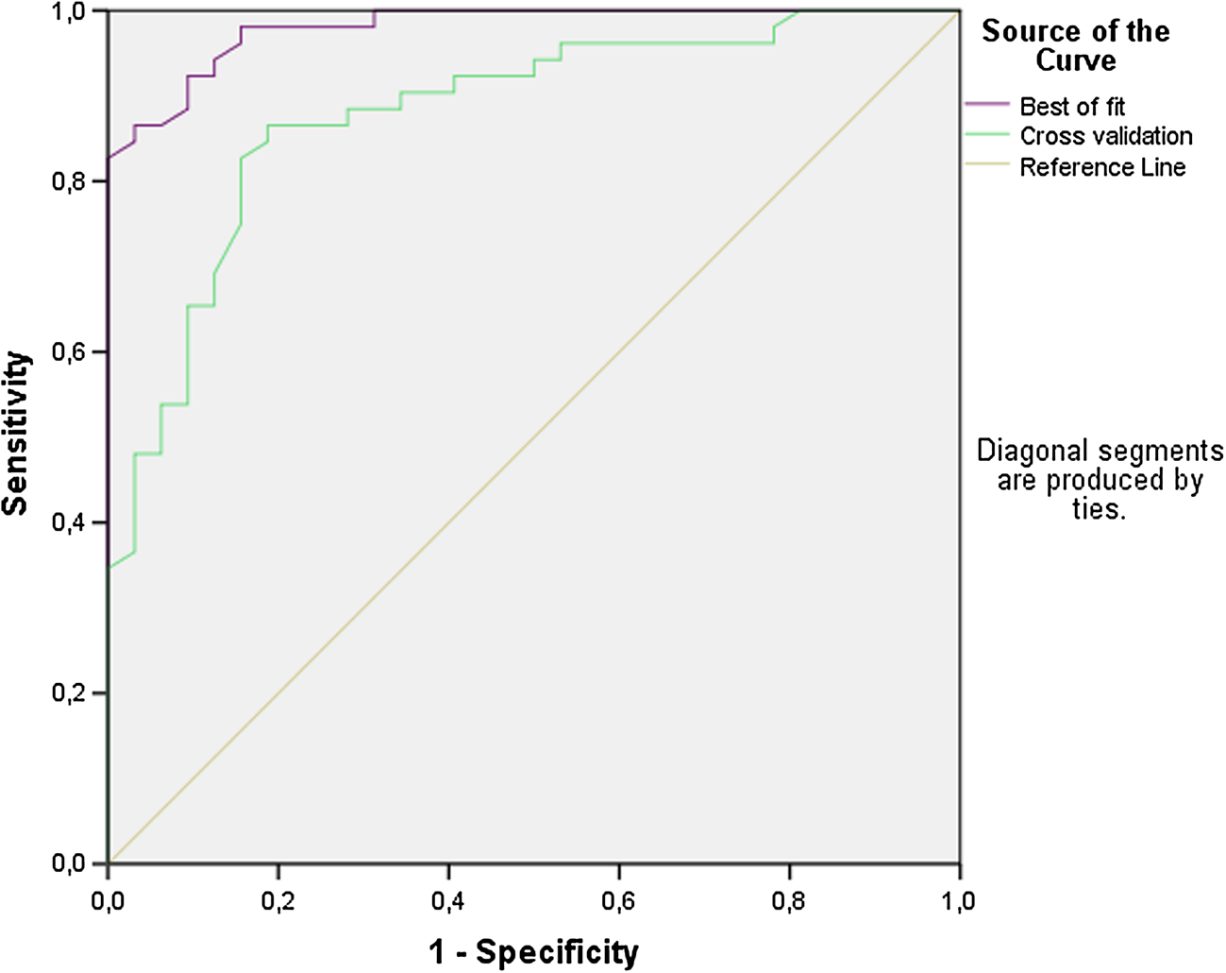
Receiver operating characteristic curve

Figure 1 displays a scatterplot of individual predictive values of a best fit of the data analyzed by the ANN. To obtain the best possible diagnostic accuracy of this data, the threshold was set to 0.55. This resulted in six patients being classified in the wrong group, with a sensitivity of 96 % and specificity of 88 %, and an overall accuracy of 93 % (MCC: 0.85) in differentiating between lung carcinoma and HNSCC. Cross-validation data is displayed in Fig. 2. The threshold for this scatterplot was set to 0.63 to obtain the best possible diagnostic accuracy. A sensitivity of 85 % and specificity of 84 % was calculated with thirteen patients being misclassified. This results in an overall accuracy of 85 % with an MCC of 0.70. The ROC-curve in Fig. 3 illustrates the different sensitivities and specificities with altered thresholds of both the best fit of the data as the cross-validation. The area under the curve (AUC) is 0.98 (95 % CI 0.96–1.00) and 0.88 (95 % CI 0.81–0.95), respectively.

## Discussion

In this proof of concept study, we have studied whether an e-nose can distinguish breath samples of patients with HNSCC from samples of patients with lung carcinoma. Given the high sensitivity and specificity for best fit of data and the leave-one-out cross-validation within this dataset, we conclude an e-nose can accurately differentiate between breath samples of patients with either tumor.

There is a growing interest in the use of VOC pattern recognition in diagnosing head and neck, and lung diseases. Chapman et al. [16] evaluated twenty patients with malignant mesothelioma with an e-nose in a cross-sectional case–control study and could successfully differentiate between mesothelioma and the 42 included healthy controls (sensitivity 90 %, specificity 91 %). D’amico et al. [17] evaluated an e-nose in patients with a vast range of lung carcinoma histopathological origins (*N* = 28) and patients with other benign lung conditions (*N* = 28) and found a correct classification of patients in 85.7 % of cases. In analyzing HNSCC, Gruber et al. [18] analyzed breath samples of 22 patients with malignant larynx and pharynx tumors, 21 patients with benign larynx and pharynx tumors, and 19 healthy controls, with an e-nose. HNSCC patients could be distinguished from healthy controls as well as from benign tumors with a sensitivity of 77 %, specificity of 90 % and overall accuracy of 83 %. In the differentiation of diseases with an e-nose, the current study adds the differentiation between two distinct oncologic entities. With an internal cross-validation of the data, we have found a sensitivity of 85 % and specificity of 84 % in differentiation HNSCC and lung carcinoma breath samples.

Although several studies report the use of an e-nose in lung or head and neck disease, different types of sensors are used in literature to observe VOC patterns, such as quartz crystal [17, 19], conducting polymers [8, 16], and metal oxide sensors [7, 9, 18], as used in the current study. This makes results of individual studies using e-nose technology hard to compare. Yet, regardless of the type of sensors used, most studies reveal promising results for VOC pattern recognition as a diagnostic tool.

VOC’s are a group of hydrocarbons such as benzene and methane. Formation of these VOC’s are found in various basic cellular functions such as oxidative stress and energy metabolism [20]. Besides that, VOC’s can originate from exogenous origin such as cigarette smoke, which can change the exhaled VOC pattern by itself or due to interaction with the human tissue [21]. With the current e-nose technique used, it remains unclear what pattern of VOC defines lung carcinoma and what defines head and neck carcinoma. Tumor growth is associated with changes in gene expression and protein changes, and associated with oxidative stress and altered metabolism. Therefore, tumor growth in general is associated with altered VOC concentrations. As mentioned earlier Peng et al. [9] revealed that different origins of cancer result in different patterns of VOC’s. This study confirms that different tumor sites result in different VOC patterns. This suggests that VOC’s produced by processes involved in tumor growth are different for other origins of cancer. The statement that different origins produce different VOC’s, might be emphasized by the increased concentrations of methylated alkanes in exhaled breath in lung cancer [22] and increased concentrations of sulfur and cyanide-containing compounds in headspace of gastric content in gastro-esophageal cancer [23].

This study indicates that the e-nose might be a valid tool in the diagnostic work-up for HNSCC or lung carcinoma. Our hypothesis is that in future clinical practice, an e-nose might be used as a tool to detect and differentiate synchronous primary lung carcinoma or metastases in patients with primary HNSCC. However, no patients were included with both primary lung carcinoma and primary HNSCC. Besides that, an e-nose might be a tool to detect primary tumors in patients with lymph node metastasis from unknown primary tumors. Although this study included only two patients with initially unknown primary lung carcinoma, both patients were correctly classified as having primary lung carcinoma. However, a larger blinded study population is necessary, to incorporate an e-nose in the diagnostic work-up for head and neck or lung carcinoma.

## Limitations

Due to the explorative character of this study, several limitations are in order. Therefor these results should be considered preliminary. A possible limitation of this study are the irregularities of the use of a neural network to calculate the predictive values of both groups. As with other statistical modalities to process large multi-way datasets such as factor analysis or principal component analysis, the model created may be partially based on artifacts in the dataset, instead of the main obvious group difference of different tumor origin. Although cross-validation revealed high sensitivity and specificity, indicating a high generalizability of the data, a large blinded dataset should be added in the future, to confirm the diagnostic accuracy of blinded data in an e-nose to differentiate between HNSCC and lung carcinoma. With this larger study population, cofactors such as history of nicotine abuse and TNM-stage can be taken into consideration in the analysis.

Some baseline characteristics were significantly different comparing both groups. The lung carcinoma groups contained mainly more advanced tumor-stages and were less often in a fasted state than the HNSCC group. Moreover, only squamous cell carcinoma patients were included in the head and neck group, whereas the lung group consists of five different histopathological origins. Although this is the natural variation in patients visiting the outpatient clinic [24], this too might influence outcomes in this dataset.

## Conclusion

This study reveals that there seems to be a potential for an e-nose as a diagnostic tool in HNSCC and lung carcinoma. With a diagnostic accuracy of 93 % and cross-validation of 85 %, an e-nose can differentiate between breath samples of patients with HNSCC and lung carcinoma. Future blinded studies with a larger study population should determine whether an e-nose can be incorporated in the diagnostic work-up for HNSCC and lung carcinoma.

## Electronic supplementary material

Below is the link to the electronic supplementary material.

Supplementary material 1 (PDF 381 kb)
