# Supplementary material for: Differentiating head and neck carcinoma from lung carcinoma with an electronic nose: a proof of concept study
Source: Eur Arch Otorhinolaryngol. 2016 Apr 16;273(11):3897–903. doi: 10.1007/s00405-016-4038-x (PMC5052311; doi:10.1007/s00405-016-4038-x)
Supplement: Supplementary file 1 — Supplementary material 1 (PDF 381 kb) [file 405_2016_4038_MOESM1_ESM.pdf]

Supplementary data 1

Patient number	Device number	Origin	Time of measurement	Age	Gender	Current smoking	Food intake <4 hours	Site	TNM-stage	Lung stage	Histopathology
1	379	Lung	15:36	60	Male	No	Yes	Left upper quadrant	pT2aN0M0	IIb	SCC
2	379	Lung	11:00	55	Female	Yes	Yes	Left lower quadrant	cT3N2M0	IIIa	Adenocarcinoma
3	379	HNC	08:31	83	Male	No	Yes	Glottic larynx	cT2N0M0		SCC
4	379	HNC	13:55	63	Male	Yes	Yes	Oropharynx	cT2N1M0		SCC
5	379	HNC	09:40	78	Male	No	No	Oral cavity	pT1N0M0		SCC
6	379	HNC	10:09	83	Female	No	Yes	Oral cavity	cT1N0M0		SCC
7	379	HNC	09:05	66	Female	Yes	Yes	Oropharynx	pT2N2aM0		SCC
8	379	Lung	15:38	58	Female	Yes	Yes	Left Hilar	cT4N2M1a	IV	SCLC
9	379	Lung	17:37	73	Male	No	Yes		cT3NxMx		Mesothelioma
10	379	HNC	11:33	60	Male	Yes	No	Oropharynx	cT2N2bM0		SCC
11	379	HNC	11:19	71	Female	No	Yes	Oral cavity	pT1N0M0		SCC
12	379	HNC	09:46	65	Male	Yes	Yes	Oral cavity	pT1N0Mx		SCC
13	379	Lung	10:32	81	Male	No	Yes	Right upper quadrant	cT4N3M1b	IV	SCLC
14	379	Lung	14:14	56	Female	Yes	Yes	Right lower quadrant	cT2aN3M1b	IV	Adenocarcinoma
15	379	HNC	13:25	85	Male	No	No	Glottic larynx	cTisN0M0		SCC
16	379	HNC	08:18	56	Male	No	No	Glottic larynx	cT1bN0M0		SCC
17	379	Lung	15:39	71	Male	No	Yes		cT3N1M0	III	Mesothelioma
18	379	Lung	15:46	54	Male	No	Yes	Left upper quadrant	cT4N2M1b	IV	Adenocarcinoma
19	362	HNC	14:45	68	Male	No	No	Glottic larynx	pT1aN0M0		SCC
20	362	HNC	09:16	81	Male	Yes	Yes	Supra glottic larynx	cT2N2cM0		SCC
21	362	HNC	09:54	60	Male	Yes	Yes	Oral cavity	pT2N0M0		SCC
22	362	HNC	10:21	92	Female	No	Yes	Oral cavity	cT2N2bM0		SCC
23	362	HNC	09:12	60	Male	Yes	Yes	Oral cavity	pT2N0M0		SCC
24	362	HNC	10:22	63	Male	Yes	Yes	Oropharynx	cT1N0M0		SCC
25	362	HNC	11:02	63	Male	Yes	No	Oropharynx	cT3N2bM0		SCC
26	362	HNC	10:13	62	Male	Yes	Yes	Oral cavity	pT3N2cM0		SCC
27	362	Lung	14:39	68	Male	Yes	Yes	Right upper quadrant	cT4N1M1a	IV	Adenocarcinoma
28	362	Lung	16:12	60	Male	Yes	Yes	Right upper quadrant	cT4N2M1b	IV	Adenocarcinoma
29	362	Lung	14:06	66	Female	Yes	Yes	Left upper quadrant	cT3N0M0	IIb	SCC

Abbreviations; HNC: head and neck carcinoma, Unkn: unknown, SCC: squamous cell carcinoma, SCLC: small cell lung carcinoma

Supplementary data 1 (continued)

Patient number	Device number	Origin	Time of measurement	Age	Gender	Current smoking	Food intake <4 hours	Site	TNM-stage	Lung stage	Histopathology
30	362	HNC	11:21	67	Male	No	Yes	Glottic larynx	cT2N0M0		SCC
31	362	Lung	13:46	66	Female	No	Yes	Right upper quadrant	cT4N2M1b	IV	SCLC
32	362	HNC	10:22	56	Male	No	Yes	Glottic larynx	cT1bN0M0		SCC
33	362	Lung	14:05	80	Male	No	Yes	Left upper quadrant	cT1N2bM0	IIIa	SCC
34	362	Lung	14:38	56	Male	Yes	Yes	Left lower quadrant	cT3N2M1b	IV	Adenocarcinoma
35	362	HNC	10.06	74	Male	No	Yes	Hypopharynx	pT2N2bM0		SCC
36	362	Lung	15:44	71	Male	No	Yes		cT3N1M0	III	Mesothelioma
37	362	Lung	16:02	54	Female	Yes	Yes	Right midquadrant	cT4N3M1b	IV	Adenocarcinoma
38	362	Lung	16:36	58	Male	Yes	Yes	Left upper quadrant	cT1bN0M0	Ia	Unknown
39	315	HNC	9:06	75	Male	No	No	Glottic larynx	cT3N0M0		SCC
40	315	HNC	12:53	57	Male	No	Yes	Oropharynx	cT3N0M0		SCC
41	315	HNC	10:53	62	Male	No	Unkn	Glottic larynx	cT1bN0M0		SCC
42	315	HNC	14:04	47	Male	Yes	No	Oral cavity	cT1N0M0		SCC
43	315	HNC	10:39	77	Male	Yes	Unkn	Glottic larynx	cT1aN0Mx		SCC
44	315	HNC	10:05	62	Female	No	Unkn	Hypopharynx	pT4aN3M0		SCC
45	315	HNC	11:14	66	Male	Yes	Yes	Oropharynx	pT1N2bM0		SCC
46	315	HNC	08:51	68	Male	Yes	Yes	Glottic larynx	pT4N1M0		SCC
47	315	HNC	09:45	51	Male	Yes	Yes	Glottic larynx	pT2N0M0		SCC
48	315	HNC	08:23	67	Female	No	Yes	Oral cavity	pT1N0M0		SCC
49	315	HNC	08:50	60	Female	Yes	Yes	Supra glottic larynx	cT3N1M0		SCC
50	315	Lung	14:23	61	Male	No	Yes	Right upper quadrant	pT1aN0M0	Ia	Neuroendocrine tumour
51	315	Lung	13:25	57	Male	Yes	Yes		cTxN2M1a	IV	Adenocarcinoma
52	315	Lung	11:04	80	Male	No	Yes	Left upper quadrant	cT1N2bM0	IIIa	SCC
53	315	Lung	12:23	56	Male	Yes	Yes	Left lower quadrant	cT3N2M1b	IV	Adenocarcinoma
54	309	HNC	10:10	51	Male	Yes	Unkn	Oropharynx	cT2N2bM0		SCC
55	309	HNC	09:01	57	Male	Yes	Unkn	Glottic larynx	cT2N2cM0		SCC
56	309	HNC	10:28	58	Male	Yes	Unkn	Oral cavity	pT2N0M0		SCC
57	309	HNC	09:45	73	Male	Yes	Unkn	Glottic larynx	cT2N0M0		SCC

Abbreviations; HNC: head and neck carcinoma, Unkn: unknown, SCC: squamous cell carcinoma, SCLC: small cell lung carcinoma

Supplementary data 1 (continued)

Patient number	Device number	Origin	Time of measurement	Age	Gender	Current smoking	Food intake <4 hours	Site	TNM-stage	Lung stage	Histopathology
58	309	HNC	09:33	57	Male	Yes	Unkn	Oral cavity	pT1N0M0		SCC
59	309	HNC	11:22	63	Male	No	Unkn	Oropharynx	cT2N0M0		SCC
60	309	HNC	09:10	78	Male	Yes	Unkn	Supra glottic larynx	cT2N1Mx		SCC
61	309	HNC	10:33	30	Male	Yes	Unkn	Oropharynx	pT2N2bM0		SCC
62	309	HNC	13:03	69	Male	Yes	Yes	Glottic larynx	pT1bN0M0		SCC
63	309	HNC	14:34	47	Male	No	No	Oral cavity	cT1N0M0		SCC
64	309	HNC	09:59	54	Male	Yes	Unkn	Hypopharynx	cT3N2bM0		SCC
65	309	HNC	08:13	30	Male	Yes	No	Oropharynx	pT2N2cM0		SCC
66	309	HNC	09:00	69	Male	Yes	Unkn	Glottic larynx	pT1bN0M0		SCC
67	309	HNC	10:11	57	Female	No	Yes	Supra glottic larynx	cT2N2bM0		SCC
68	309	Lung	16:27	66	Female	Yes	Yes		cTxN3M1b	IIIb	Adenocarcinoma
69	309	Lung	10:59	73	Male	No	Yes	Left upper quadrant	cT4aN2M1b	IV	Adenocarcinoma
70	259	Lung	14:15	78	Female	No	Yes	Left lower quadrant	cT3N2M1b	IV	Adenocarcinoma
71	259	Lung	10:18	62	Female	No	No	Left lower quadrant	pT1aN2M0	IIIa	Adenocarcinoma
72	259	Lung	09:58	77	Male	No	Yes	Right upper quadrant	cT4N2M1b	IV	SCC
73	259	Lung	09:38	67	Male	No	No	Right upper quadrant	pT3N1M0	IIIa	Neuroendocrine tumour
74	259	Lung	15:48	69	Female	No	Yes	Right lower quadrant	cT4N2M0	IIIb	Adenocarcinoma
75	259	Lung	11:45	80	Male	No	Yes	Right lower quadrant	pT1bN0M0	Ia	Adenocarcinoma
76	259	Lung	13:15	46	Female	Yes	Yes	Right upper quadrant	cT3N2M0	IIIa	Adenocarcinoma
77	259	Lung	14:28	63	Male	No	Yes	Left lower quadrant	cT3N3N0	IIIb	Adenocarcinoma
78	259	Lung	14:03	56	Female	No	Yes	Right upper quadrant	T2aN2M1b	IV	Adenocarcinoma
79	259	HNC	10:18	72	Male	Yes	Yes	Oropharynx	cT2N0M0		SCC
80	259	HNC	14:23	63	Male	Yes	No	Oropharynx	cT1N0M0		SCC
81	259	HNC	14:29	62	Male	Yes	Yes	Oral cavity	pT3N2cM0		SCC
82	259	HNC	10:20	34	Male	Yes	Yes	Cavum nasi	cT3N3bM1		SCC
83	259	HNC	11:27	46	Male	Yes	Yes	Oral cavity	pT2N2bM0		SCC
84	259	HNC	09:16	51	Female	No	Yes	Cavum nasi	cT2N0M0		SCC

Abbreviations; HNC: head and neck carcinoma, Unkn: unknown, SCC: squamous cell carcinoma, SCLC: small cell lung carcinoma
